# A Two-Stage Interpretable Fault Diagnosis Approach for Bearings Based on EBM

**DOI:** 10.3390/s26123679

**Published:** 2026-06-09

**Authors:** Suyi Zheng, Dajun Li, Mingxuan Xiong, Meng Yang, Fanqiang Lin

**Affiliations:** 1 College of Computer Science and Cyber Security, Chengdu University of Technology, Chengdu 610059, China; 202313160528@stu.cdut.edu.cn (S.Z.); xiongmingxuan@stu.cdut.edu.cn (M.X.); 2 College of Mechanical and Electrical Engineering, Chengdu University of Technology, Chengdu 610059, China; linfq@cdut.edu.cn; 3 School of Computer Science and Engineering, University of Electronic Science and Technology of China, Chengdu 610059, China; 202521080903@std.uestc.edu.cn

**Keywords:** bearing fault diagnosis, interpretable machine learning, feature selection, random forest, explainable boosting machine

## Abstract

In recent years, explainable artificial intelligence has received increasing attention in the field of bearing fault diagnosis. However, existing interpretability methods, such as Shapley Additive Explanations (SHAP), often rely on the quality of input features. To achieve high diagnostic accuracy, researchers often extract a large number of features from vibration signals across multiple domains, leading to feature redundancy. This redundancy not only increases the computational cost and risk of overfitting in diagnostic models but also dilutes the contributions of core features during interpretability analysis, resulting in biased explanations. To address this challenge, we propose a two-stage interpretable fault diagnosis approach. In the first stage, the Explainable Boosting Machine (EBM) selects core features to reduce redundancy. In the second stage, EBM is enhanced by Random Forest (RF) through residual learning to form the RF-EBM diagnostic model. EBM and SHAP are further used for dual interpretability analysis. Experimental results on public laboratory benchmark datasets demonstrate that the proposed approach achieves good diagnostic performance and outperforms traditional EBM. Overall, the approach reduces redundancy through feature selection, improves diagnostic performance, and makes the decision-making process more transparent, providing a useful methodological reference for trustworthy fault diagnosis in industrial applications.

## 1. Introduction

Currently, bearings are essential for the safe and stable operation of modern industrial power systems. In electric motors, they support rotating loads and reduce friction, while often operating at high speeds, under variable loads, and in complex lubrication conditions [1]. These demanding conditions make bearings susceptible to fatigue spalling, indentations, wear, etc. [2]. Bearing faults are the most common type of fault in induction motors, accounting for approximately 40% of motor failures [3]. Such faults can cause severe vibration and noise and may further affect the shaft system, resulting in stator rubbing, shaft deformation, or even complete machine failure [4]. Therefore, timely and accurate bearing fault diagnosis supports predictive maintenance and helps reduce economic losses and safety risks.

The development of bearing fault diagnosis methods has long been plagued by the paradox between accuracy and interpretability [5]. Although many black-box models achieve high diagnostic accuracy, their decision-making processes are difficult to trace. In practical applications, when false positives or missed detections occur, this lack of interpretability makes it difficult to determine why a diagnosis is incorrect and how it can be corrected. This trade-off undermines the reliability of diagnostic results and weakens the confidence of field engineers, thereby hindering the transition from laboratory research to practical applications [6,7]. The evolution of diagnostic methods illustrates this paradox. Early research relied on physics-based analytical methods and amplitude-threshold alarms [8]. These approaches followed clear rules and were easy to understand, but they had limited ability to capture nonlinear vibration behavior under varying loads and speeds, resulting in relatively low diagnostic accuracy [9,10]. With the development of machine learning techniques (e.g., SVM, KNN, and MLP) [11], diagnostic accuracy improved through the incorporation of larger feature sets, often including more than ten features extracted from multiple domains [12]. Nevertheless, this increase in feature dimensionality introduced a new challenge. Although these models improved diagnostic performance, high dimensionality and feature redundancy increased the complexity of the decision-making process. As a result, the internal logic of the models remained opaque, compromising interpretability and reducing the trustworthiness of diagnostic outcomes.

To address this issue, recent research has introduced post-hoc explanation methods (e.g., Shapley Additive Explanations (SHAP) and Local Interpretable Model-agnostic Explanations (LIME)) [13,14,15,16], which estimate each feature’s contribution to model predictions. Nonetheless, the effectiveness of these methods still depends on the quality of the input features [17]. Redundant features may dilute the contributions of core features, causing important discriminative features to be ranked lower [18]. Researchers have attempted to address this problem by adopting methods such as mutual information and principal component analysis for feature selection [19,20]. However, mutual information mainly relies on a single relevance criterion and may overlook feature interactions, while principal component analysis does not use label information and generates transformed features that are difficult to interpret [21,22].

To tackle the aforementioned issues, we propose a two-stage interpretable diagnostic approach that aims to maintain high diagnostic accuracy while improving the interpretability of the diagnostic process. In the first stage, EBM is employed for feature selection owing to its interpretable additive structure and its ability to model nonlinear feature effects. EBM quantifies the contributions of individual features and pairwise interactions, thereby providing a transparent basis for identifying fault-sensitive features. By incorporating fault label information, EBM ranks features according to their diagnostic contributions, enabling the selection of a compact set of core features [23]. Focusing subsequent interpretability analysis on these core features reduces redundancy and makes the explanation process clearer and more reliable.

In the second stage, EBM serves as the primary interpretable diagnostic model for fault diagnosis. However, as its additive structure mainly represents individual feature effects and pairwise interactions between features, EBM may not fully capture higher-order nonlinear relationships among fault-sensitive features [24]. Thus, residual learning is introduced to compensate for the prediction bias that may remain in the EBM output. Specifically, Random Forest (RF) learns residual correction information and incorporates it into the EBM prediction through a confidence-weighted fusion strategy. In this design, RF acts as a residual enhancer, while EBM remains the primary interpretable model. The decision-making process is then interpreted using EBM-based analysis for the primary model and SHAP for the RF enhancement component. Our main contributions are threefold:

1. An EBM-based feature selection method is proposed, which identifies core features to reduce feature redundancy, decreases model complexity, and simplifies interpretability analysis.
2. An explainable diagnostic model, RF-EBM, is constructed. It introduces RF through residual learning to enhance EBM and improve diagnostic accuracy.
3. A dual interpretability strategy based on EBM and SHAP is established to support transparent decision analysis and improve model interpretability.

## 2. Methodology

**Figure 1:**
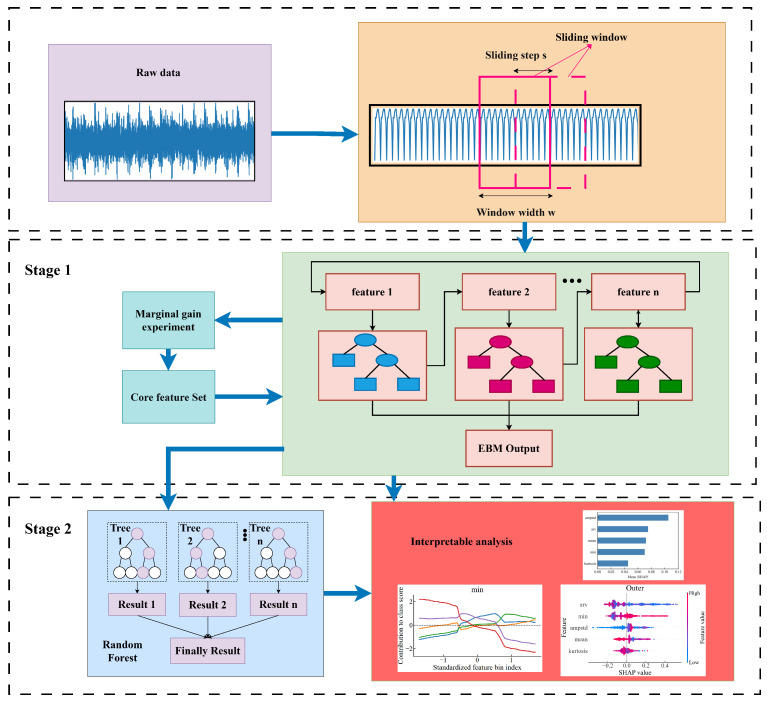
Workflow of the proposed two-stage interpretable diagnostic approach.

The proposed approach follows a two-stage interpretable diagnostic framework: EBM-based core feature selection and RF-EBM diagnostic modeling. Before these two stages, vibration signals are segmented into fixed-length samples and converted into feature vectors. In the first stage, EBM is used to evaluate feature contributions and select the core feature set. In the second stage, the selected features are used to train the RF-EBM model, in which RF learns residual correction information to refine the EBM prediction. The workflow is shown in Figure 1.

### 2.1. Signal Sample Construction

**Figure 2:**
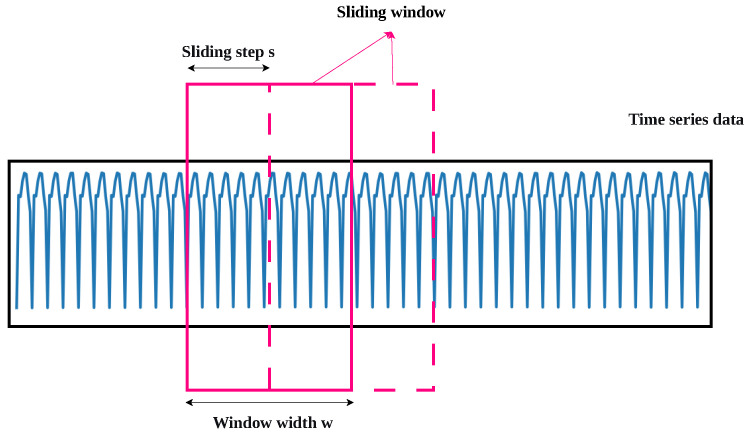
Schematic diagram of the sliding window segmentation process.

Bearing vibration signals are continuous time-series data acquired by sensors. To construct standardized samples for feature extraction, feature selection, and model comparison, this study employs an overlapping sliding window segmentation method to preprocess the raw signals. The segmentation process is shown in Figure 2. The main characteristics of this method are as follows:

1. It reduces the possibility that fault-related impulse components are truncated at segment boundaries, thereby improving the representation of fault information within each sample.
2. It uses overlapping sampling to increase the number of available samples, providing sufficient data support for subsequent EBM-based feature selection [25].
3. It improves data utilization while maintaining computational efficiency, achieving a reasonable balance between information redundancy and processing speed.

The sliding window method is controlled by two parameters: the window width *w* and the sliding step size *s*. Here, *w* determines the number of sampling points included in each sample, while *s* determines the overlap between adjacent windows. For a continuous time series of length *L*, the total number of window samples *n* is calculated as follows:

(1) $$n=\left\lfloor \frac{L-w}{s}\right\rfloor +1$$

### 2.2. Feature Extraction from Vibration Signals

A total of 23 initial features are extracted from the raw vibration signals, covering commonly used time-domain and frequency-domain features in bearing fault diagnosis. These features characterize the vibration signals under different bearing conditions. The specific features are listed in Table 1 and Table 2.

### 2.3. Construction of the RF-EBM Model

#### 2.3.1. Model Architecture

RF-EBM consists of three core components: an EBM primary model, an RF residual enhancer, and a confidence-weighted fusion module. During diagnosis, EBM first outputs the predicted probabilities for all fault classes, while RF provides residual correction information based on the input features. The final diagnosis is then obtained by fusing the EBM output and RF residual correction through the confidence-weighted module.

#### 2.3.2. Overview of EBM

The mathematical formulation of EBM is based on the Generalized Additive Model (GAM). In this structure, the model output is expressed as the sum of a baseline term, individual feature effects, and pairwise interaction effects:

(2) $$g\left(E\left[y\right]\right)=\beta_{0}+\sum_{j=1}^{d}f_{j}\left(x_{j}\right)+\sum_{i<j}f_{ij}\left(x_{i},x_{j}\right)$$

where g(·) denotes the link function, E[y] is the expected value of the target variable, and $\beta_{0}$ represents the baseline model output. The function $f_{j}\left(x_{j}\right)$ denotes the main effect of the *j*-th feature, indicating the contribution of this feature to the model output when its value is $x_{j}$. Here, *d* is the total number of input features. The term $f_{ij}\left(x_{i},x_{j}\right)$ represents the pairwise interaction effect between the *i*-th and *j*-th features, indicating the additional contribution produced by their joint effect. The summation over i<j ensures that each feature pair is considered only once.

The interpretability of EBM stems from this additive decomposition. Since the model output is separated into main effects and pairwise interaction effects, each contribution can be isolated and visualized through shape functions. These shape functions show how different feature values affect the model output. By aggregating these contributions across samples, EBM produces global importance scores and ranks features according to their overall contribution magnitudes. This ranking provides a transparent basis for feature selection, allowing the most diagnostically relevant features to be retained for subsequent modeling and interpretability analysis.

The above equation describes the general additive structure of EBM. In the bearing fault diagnosis task, the model output corresponds to a multi-class bearing condition label. Therefore, EBM learns a separate additive score for each fault class, and these class scores are converted into class probabilities using the softmax function:

(3) $$P_{\mathrm{EBM}}\left(y=k|x\right)=\frac{\exp\left(\beta_{0,k}+\sum_{j=1}^{d}f_{j,k}\left(x_{j}\right)+\sum_{i<j}f_{ij,k}\left(x_{i},x_{j}\right)\right)}{\sum_{\ell =1}^{C}\exp\left(\beta_{0,\ell }+\sum_{j=1}^{d}f_{j,\ell }\left(x_{j}\right)+\sum_{i<j}f_{ij,\ell }\left(x_{i},x_{j}\right)\right)}$$

where $P_{\mathrm{EBM}}\left(y=k|x\right)$ denotes the probability predicted by EBM that sample *x* belongs to fault class *k*, and *C* is the total number of fault classes. In the numerator, $\beta_{0,k}$ represents the baseline score of class *k*, which is the initial class score before considering the contribution of specific feature values. The term $f_{j,k}\left(x_{j}\right)$ represents the contribution of the *j*-th feature to the score of class *k* when this feature takes the value $x_{j}$. The term $f_{ij,k}\left(x_{i},x_{j}\right)$ represents the additional contribution produced by the interaction between the *i*-th and *j*-th features for class *k*. In the denominator, the same additive score is computed for each class *ℓ* and normalized over all fault classes through the softmax function, so that the predicted probabilities sum to one.

#### 2.3.3. Overview of Residual Learning and Random Forest

As an additive model, EBM can effectively capture main feature effects and pairwise interactions. However, higher-order nonlinear relationships may still exist among fault-sensitive features, and these relationships may not be fully captured by the additive structure of EBM. Therefore, a residual learning mechanism is introduced to compensate for the prediction bias of EBM.

The residual captures the discrepancy between the true label and the EBM prediction, reflecting the prediction bias of EBM across different bearing conditions [26]. For each training sample, EBM first outputs the predicted probability for each fault class, while the true label is represented using one-hot encoding. For the *k*-th class, the residual is defined as

(4) $$r^{\left(k\right)}\left(x\right)=e^{\left(k\right)}-P_{\mathrm{EBM}}\left(y=k|x\right)$$

where $r^{\left(k\right)}\left(x\right)$ denotes the residual for the *k*-th class, $e^{\left(k\right)}$ is the one-hot label value of the *k*-th class, and $P_{\mathrm{EBM}}\left(y=k|x\right)$ is the probability predicted by EBM for the *k*-th class. A positive $r^{\left(k\right)}\left(x\right)$ indicates that EBM underestimates the probability of the corresponding class, whereas a negative value indicates overestimation.

Considering both model characteristics and comparative experimental results, RF is selected as the residual learner in this study. After feature selection using EBM, the original features from multiple domains are reduced to a small set of features that are sensitive to faults, forming a tabular input space with low dimensionality. RF is suitable for this type of input because it can model nonlinear relationships and feature interactions without requiring strong assumptions about feature distributions. Its ensemble structure can reduce the influence of unstable splits from individual decision trees and improve prediction stability [27,28,29]. Therefore, RF learns residual correction information from the selected features and EBM prediction residuals. The learned correction information is then used to compensate for the EBM output in the subsequent confidence-weighted fusion stage.

The residuals defined above are used to train RF as a residual learner. In this setting, RF learns the relationship between the selected features and the residual correction information derived from the EBM prediction errors. For a given sample *x* and fault class *k*, the RF residual correction output is obtained by aggregating the outputs of multiple decision trees:

(5) $$g_{\mathrm{RF}}^{\left(k\right)}\left(x\right)=\frac{1}{M}\sum_{m=1}^{M}T_{m}^{\left(k\right)}\left(x\right)$$

where $g_{\mathrm{RF}}^{\left(k\right)}\left(x\right)$ denotes the RF residual correction output for fault class *k*, *M* is the total number of decision trees, and $T_{m}^{\left(k\right)}\left(x\right)$ denotes the output of the *m*-th decision tree for class *k*. This output represents the residual correction score estimated by RF and is subsequently used in the confidence-weighted fusion stage to compensate for the EBM probability output.

#### 2.3.4. Confidence-Weighted Fusion Strategy

After obtaining the residual correction output from RF, a fusion strategy based on EBM confidence is used to combine it with the primary EBM prediction. In this strategy, EBM provides the primary class probability output, RF provides the residual correction output, and the EBM confidence score determines the enhancement strength of the RF correction.

The confidence score is defined as the maximum predicted probability among all fault classes:

(6) $$c=\max_{k}P_{\mathrm{EBM}}\left(y=k|x\right)$$

where *c* denotes the confidence score, $P_{\mathrm{EBM}}\left(y=k|x\right)$ is the probability predicted by EBM that sample *x* belongs to fault class *k*, and *k* ranges over all fault classes. A higher confidence score indicates that EBM assigns a dominant probability to one fault class, whereas a lower confidence score indicates that the predicted probabilities are more evenly distributed across classes.

Based on the confidence score, a piecewise fusion rule is used to determine the enhancement strength λ. A smaller enhancement strength is assigned to high confidence predictions to avoid excessive correction, while a larger enhancement strength is assigned to low-confidence predictions to increase the contribution of the RF residual correction. The confidence segmentation and enhancement strength were determined through a validation-based fusion parameter optimization experiment, which jointly optimized the number of confidence segments, confidence thresholds, and enhancement strength on the validation set. These parameters can be recalibrated when the dataset, signal distribution, or operating conditions change.

The final prediction of RF-EBM is obtained by combining the EBM primary probability output with the residual correction output from RF at the score level. For fault class *k*, the corrected class score is defined as

(7) $$S^{\left(k\right)}\left(x\right)=P_{\mathrm{EBM}}\left(y=k|x\right)+\lambda \cdot g_{\mathrm{RF}}^{\left(k\right)}\left(x\right)$$

where $S^{\left(k\right)}\left(x\right)$ denotes the corrected score for fault class *k*, $P_{\mathrm{EBM}}\left(y=k|x\right)$ is the EBM probability output, λ is the enhancement strength determined by the confidence level, and $g_{\mathrm{RF}}^{\left(k\right)}\left(x\right)$ is the residual correction output from RF. It should be noted that $S^{\left(k\right)}\left(x\right)$ is a corrected class score rather than a normalized probability. The fault class with the highest corrected score is selected as the final diagnostic result.

#### 2.3.5. Interpretability Methods

To improve the interpretability of the RF-EBM decision-making process, two interpretation methods are used in this study. The EBM shape functions described in Section 2.3.2 explain the decision logic of the primary EBM model, while SHAP analysis is used to interpret the RF residual enhancer.

SHAP is a post-hoc interpretability method based on cooperative game theory. It quantifies the contribution of each feature to the model prediction by computing the weighted average of its marginal contributions across different feature subsets [13]. The SHAP value $\phi_{i}$ for feature *i* is defined as

(8) $$\phi_{i}=\sum_{S\subseteq N\setminus \{i\}}\frac{|S|!\;(|N|-|S|-1)!}{|N|!}\left[f(S\cup \{i\})-f(S)\right]$$

where *N* is the set of all features, *S* is a feature subset that does not contain feature *i*, and f(S) denotes the model output when only the features in *S* are considered. The term f(S∪{i})−f(S) represents the marginal contribution of feature *i*. In this study, SHAP is applied to the RF residual enhancer to explain the contribution of each core feature to the residual correction output for each fault class.

By comparing the interpretation results of EBM and SHAP, the consistency of feature contributions can be examined from two perspectives. If the two methods identify similar important features, it suggests that the primary EBM model and the RF residual enhancer rely on similar diagnostic information, making the diagnostic basis more reliable. If their results differ, the difference should be interpreted as complementary information rather than a direct conflict, because EBM explains the primary prediction structure, whereas SHAP explains the residual correction component. In such cases, the analysis should focus on low-confidence samples, misclassified samples, and samples with large RF correction scores to determine whether the different feature contributions are associated with the residual compensation introduced by RF.

### 2.4. Performance Evaluation Metrics

To facilitate fair comparison and comprehensive evaluation, this study adopts Accuracy, Precision, Recall, F1 score, AUC, and inference time as evaluation metrics [30,31]. The first four are defined as follows:

(9) $$\mathrm{Accuracy}=\frac{TP+TN}{TP+TN+FP+FN}$$

(10) $$\mathrm{Precision}=\frac{TP}{TP+FP}$$

(11) $$\mathrm{Recall}=\frac{TP}{TP+FN}$$

(12) $$F1=2\times \frac{\mathrm{Precision}\times \mathrm{Recall}}{\mathrm{Precision}+\mathrm{Recall}}$$

where TP, TN, FP, and FN denote true positives, true negatives, false positives, and false negatives, respectively.

AUC measures the probability ranking quality. Inference time reflects computational efficiency. For multi-class tasks, Precision, Recall, F1, and AUC are macro-averaged.

The computational experiments were performed on a computer equipped with a 13th Gen Intel(R) Core(TM) i7-1355U processor and 16 GB RAM. The reported inference time represents the average time per sample.

## 3. Results

### 3.1. Dataset and Preprocessing

**Figure 3:**
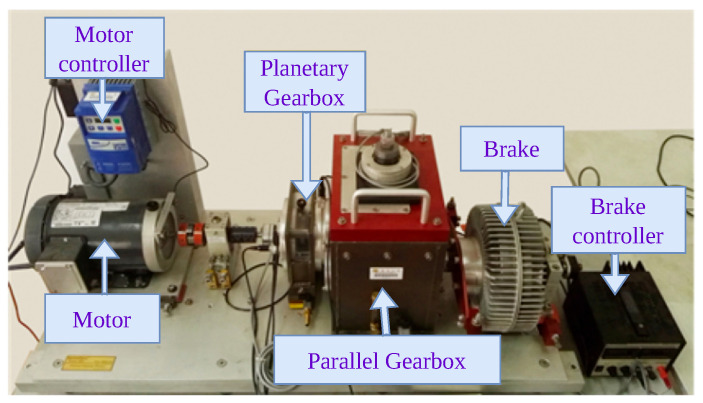
Experimental setup of the SEU bearing fault diagnosis test bench.

The motor vibration data used in this study are sourced from the Southeast University (SEU) bearing fault diagnosis dataset [32]. The experimental setup is shown in Figure 3. It consists of six components: a motor controller, a motor, a planetary gearbox, a parallel gearbox, a brake, and a brake controller. Seven 608A11 vibration sensors (PCB Piezotronics, Depew, NY, USA) were installed to collect data along the *x*, *y*, and *z* axes of the planetary and parallel gearboxes, as well as the *z*-axis of the motor, at a sampling frequency of 5120 Hz. The test bench was configured for two typical operating conditions: a motor speed of 1200 rpm with a load of 0 Nm and a motor speed of 1800 rpm with a load of 7.32 Nm. The bearing condition types are listed in Table 3 [33,34].

To obtain stable diagnostic features while maintaining a sufficient number of samples, the sliding window method uses a window width of 1024 points and a step size of 512 points, resulting in a 50% overlap. These values have also been adopted in many bearing fault diagnosis studies based on vibration signals [35]. The 1024-point window provides a sufficiently long signal segment for feature extraction and is computationally convenient for FFT-based frequency-domain analysis. The 50% overlap increases the number of available samples and reduces the possibility that fault-related impacts are truncated at the window boundary. The dataset is then randomly split, with 70% of the samples used for training and 30% used for testing.

### 3.2. Core Feature Selection

To select the core features, EBM was used for feature contribution analysis. The EBM parameters were determined through preliminary experiments to obtain stable feature importance rankings and reliable diagnostic performance under the current dataset. The final parameter settings are shown in Table 4.

A total of 23 features were extracted from the raw signals and initially used as inputs to EBM for interpretable modeling. This allowed EBM to learn the contribution pattern of each feature while performing fault classification.

**Figure 4:**
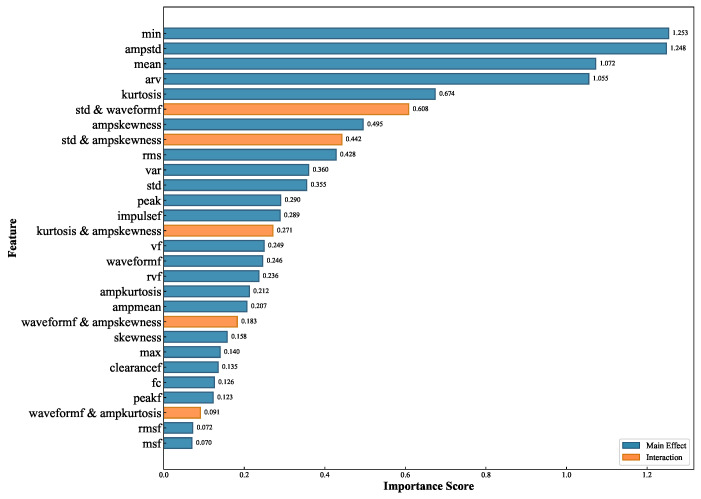
Feature importance ranking obtained from EBM. Main effects indicate individual feature contributions, whereas interaction terms indicate joint contributions between feature pairs.

As shown in Figure 4, the feature importance ranking generated by EBM includes both main effects and pairwise interaction terms.

**Figure 5:**
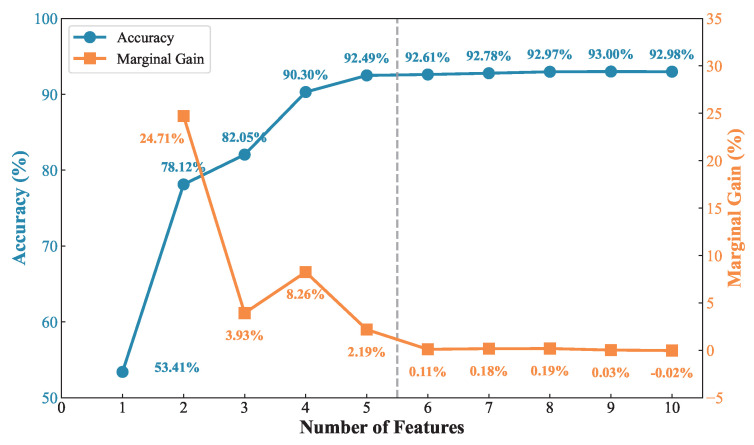
Marginal gain of accuracy with respect to the number of features.

To determine the optimal number of features, a marginal gain analysis was conducted. Features were added one by one in descending order of EBM importance, and EBM was retrained after each addition to record the corresponding classification accuracy. The results are shown in Figure 5.

When the number of features increased from one to five, the accuracy rose rapidly from 53.41% to 92.49%, with a total gain of 39.08%. The marginal gain obtained by adding the fifth feature, kurtosis, was 2.19%. After adding the sixth feature, std, the accuracy improved by only 0.11%. The subsequent features yielded marginal gains of 0.18%, 0.19%, 0.03%, and −0.02%, respectively. These results indicate that the first five features capture most of the discriminative information, whereas additional features provide negligible improvements and may even slightly degrade performance. Therefore, min, ampstd, mean, arv, and kurtosis were selected as the core feature set.

The formulas of the five core features are as follows:

(13) $$\min=\min_{1\leq i\leq w}x_{i}$$

(14) $$\mathrm{ampstd}=\sqrt{\frac{1}{N_{f}}\sum_{q=1}^{N_{f}}{\left(B_{q}-\bar{B}\right)}^{2}}$$

(15) $$\mathrm{mean}=\frac{1}{w}\sum_{i=1}^{w}x_{i}$$

(16) $$\mathrm{arv}=\frac{1}{w}\sum_{i=1}^{w}\left|x_{i}\right|$$

(17) $$\mathrm{kurtosis}=\frac{1}{w}\sum_{i=1}^{w}{\left(\frac{x_{i}-\bar{x}}{\sigma }\right)}^{4}-3$$

where $x_{i}$ is the *i*-th sampling point within a signal window, and *w* is the window width, namely the number of sampling points in each window. $\bar{x}$ is the mean value of the signal window, and σ is the standard deviation of the signal window. $B_{q}$ denotes the spectral amplitude at the *q*-th frequency bin, $\bar{B}$ is the mean spectral amplitude, and $N_{f}$ is the total number of frequency bins.

To validate the effectiveness of the proposed feature selection method, a comparative experiment was conducted. Five random feature sets were generated from the original 23 features, with each set containing five features. The composition of each random feature set is shown in Table 5. Each feature set was then used as the input to EBM for modeling, and its diagnostic performance was compared with that of the selected core feature set.

The same EBM parameter settings and training data are used across all experiments to ensure a fair comparison. The evaluation metrics include Accuracy and F1 score. The results are shown in Table 6.

The results suggest that the core feature set obtained through EBM captures more fault-related information than the random feature sets under the current experimental setting. As shown in Table 6, the core feature set achieves the highest accuracy (92.49%) and F1 score (92.45%), outperforming all five random feature sets.

### 3.3. Model Comparison

To evaluate the applicability of the selected core feature set across different model types and to support the selection of the residual learner, five algorithms were compared: SVM, XGBoost, KNN, MLP, and RF.

The diagnostic performance of these models on the core feature set is reported in Table 7. RF, KNN, MLP, and XGBoost all achieved accuracy values above 93%, whereas SVM achieved an accuracy of 81.78%. The lower performance of SVM suggests that, under the current experimental setting, SVM has a weaker ability to capture the discriminative information contained in the selected features [36]. In contrast, RF, XGBoost, and MLP are more capable of modeling nonlinear relationships and, therefore, can better capture the fault-related information contained in the core features. Among the five models, RF achieved the highest accuracy, precision, recall, and F1 score, with values of 94.63%, 94.63%, 94.63%, and 94.62%, respectively, while XGBoost achieved the highest AUC of 99.65%. Although XGBoost showed a slightly higher AUC, RF provided better overall diagnostic performance on the core feature set. Therefore, together with its suitability as a residual learner discussed above, RF was selected for constructing the RF-EBM model.

### 3.4. Design of RF-EBM

The main parameter settings of the RF residual learner are shown in Table 8. These parameters were selected according to the characteristics of the small core feature set and the role of RF as a residual learner. A relatively large number of decision trees was used to obtain stable ensemble outputs, while bootstrap sampling and the square root rule for feature selection at each split were adopted to increase tree diversity. The splitting settings allow the trees to capture nonlinear residual correction patterns from the selected features without introducing unnecessary parameter complexity.

**Figure 6:**
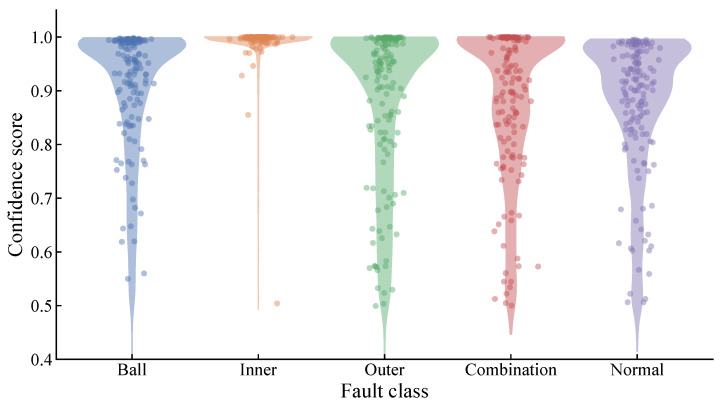
Confidence score distribution of EBM predictions across different bearing conditions.

The enhancement rule of RF-EBM consists of two key elements: confidence segmentation thresholds and enhancement strengths assigned to each interval. Figure 6 shows the confidence score distribution of EBM predictions across the five bearing conditions. The distribution indicates that EBM predictions are generally more reliable in regions with high confidence, whereas samples with lower confidence correspond to more uncertain predictions. Therefore, a piecewise fusion strategy is adopted to assign different enhancement strengths according to the confidence level.

The confidence thresholds and enhancement strengths were determined through a fusion parameter tuning experiment based on validation data. Classification accuracy was used as the primary optimization criterion, while the F1 score was used as an auxiliary reference. According to the tuning results, the confidence thresholds were set to 0.95, 0.90, 0.85, and 0.80, dividing the EBM confidence range into five intervals. The enhancement strength was set to be inversely related to EBM confidence: higher confidence predictions receive weaker RF residual correction to avoid overcorrection, whereas lower confidence predictions receive stronger correction to compensate for greater uncertainty. The selected values were obtained from validation experiments conducted on the datasets used in this study and can be recalibrated when the dataset, signal distribution, or operating conditions change. The final piecewise function is defined as follows:

(18) $$\lambda =\phi \left(c\right)=\left\{\begin{array}{cc} 0.6, & c>0.95\\ 1, & 0.90<c\leq 0.95\\ 1.5, & 0.85<c\leq 0.90\\ 2, & 0.80<c\leq 0.85\\ 2.5, & c\leq 0.80 \end{array}\right.$$

To validate the effectiveness of the RF-EBM model, an ablation experiment was conducted by comparing RF-EBM with traditional EBM on the core feature set.

**Figure 7:**
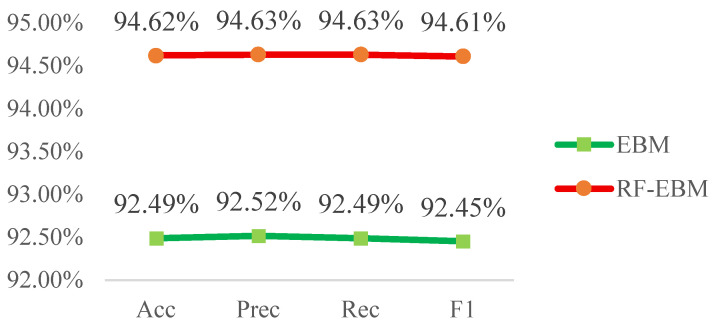
Diagnostic performance comparison of traditional EBM and RF-EBM.

As shown in Figure 7, RF-EBM improves the diagnostic accuracy from 92.49% to 94.62%, corresponding to an increase of 2.13 percentage points over traditional EBM. The F1 score increases from 92.45% to 94.61%, with an improvement of 2.16 percentage points. Precision and recall also show similar improvements. These results indicate that learning the prediction residuals of EBM through RF and incorporating them through a fusion rule based on confidence can further improve diagnostic performance. Therefore, RF-EBM compensates for part of the prediction bias of EBM while retaining EBM as the primary interpretable model.

### 3.5. Interpretability Analysis

**Figure 8:**
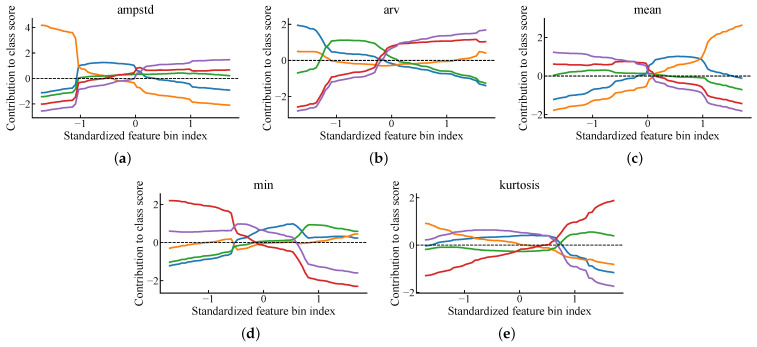
Shape function plots of the five core features: (**a**) ampstd, (**b**) arv, (**c**) mean, (**d**) min, and (**e**) kurtosis. In each subplot, the colored curves represent feature contribution to different classes: Ball (blue), Inner (orange), Outer (green), Combination (red), and Normal (purple). The horizontal axis represents the standardized feature bin index, and the vertical axis represents the contribution to the class score. Positive values increase the class score, whereas negative values decrease it.

The EBM shape function plots are shown in Figure 8. These plots illustrate how each core feature influences the model output for different bearing conditions. Taking ampstd as an example, as its value increases from low to high, its contribution to Inner faults changes from positive to negative. For Combination and Normal conditions, the contribution changes from negative to positive. For Outer faults, the contribution changes from negative to slightly positive, whereas for Ball faults, it first changes from negative to positive and then decreases again. These class-specific contribution patterns help explain the decision logic of EBM and can support the analysis of misclassified samples.

**Figure 9:**
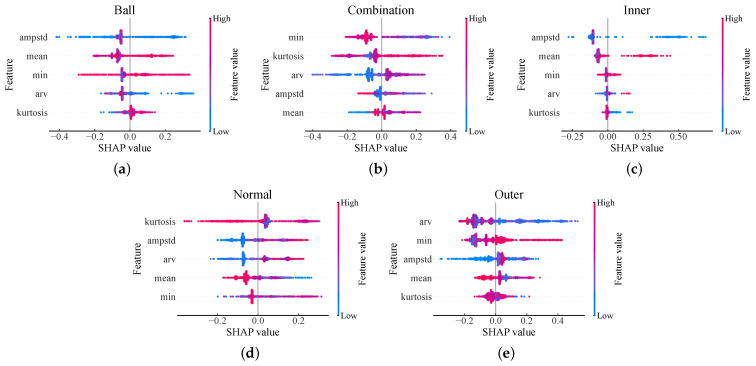
SHAP beeswarm plots of the RF residual enhancer: (**a**) Ball, (**b**) Combination, (**c**) Inner, (**d**) Normal, and (**e**) Outer. Each point represents one sample. SHAP values on the right indicate positive contributions, while values on the left indicate negative contributions. Color indicates the feature value, with red for high values and blue for low values. Features are ordered by global importance.

The SHAP analysis results of the RF residual enhancer are shown in Figure 9. For Ball and Inner faults, the RF residual correction is mainly associated with ampstd, suggesting that this feature contributes more to the correction of these two fault categories. For Combination faults, min shows a relatively larger contribution. For Outer faults, arv and min contribute more prominently, which may be associated with the relatively stable impact amplitude of this fault type. For Normal conditions, kurtosis shows a larger contribution to the residual correction output.

Taking Figure 9a as an example, ampstd is ranked as the most important feature for Ball faults and shows a negative association with the RF correction output, indicating that lower ampstd values tend to correspond to higher SHAP values for this class. The second- and third-ranked features, mean and min, show positive associations, where higher feature values tend to increase the RF correction output for Ball faults. In contrast, arv shows a negative association, with lower values corresponding to higher SHAP values. Kurtosis has a relatively weak positive influence. Overall, the SHAP results suggest that ampstd, min, and mean provide the main contribution to the RF residual correction for Ball fault predictions.

**Figure 10:**
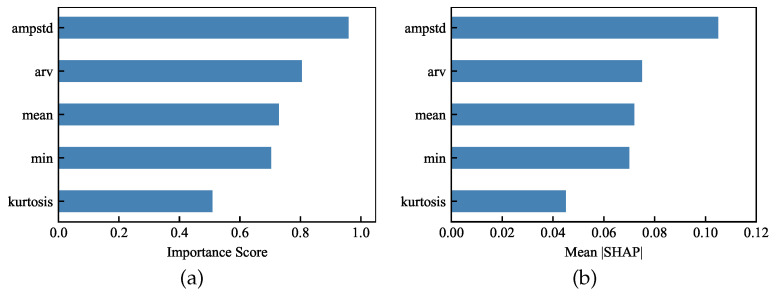
Core feature importance rankings obtained from two interpretation perspectives: (**a**) EBM primary model, (**b**) SHAP analysis of the RF residual enhancer.

Figure 10 compares the feature importance rankings obtained from two interpretation perspectives. The two rankings are consistent, with ampstd ranked first in both methods, followed by arv, mean, min, and kurtosis. As discussed in Section 2.3.5, when different interpretation methods produce consistent feature rankings, the diagnostic basis of the model can be considered more reliable. This consistency indicates that the RF residual enhancer is aligned with the EBM primary model in terms of feature contribution, and the combined interpretation provides a more reliable basis for understanding the diagnostic decision-making process.

### 3.6. Validation on the CWRU Dataset

To evaluate the applicability of the proposed approach, the proposed approach was validated on the Case Western Reserve University (CWRU) dataset [37]. The drive-end vibration signals sampled at 12 kHz were used. This dataset contains four operating conditions, and each condition corresponds to a specific speed and load combination. For this dataset, the EBM-based feature selection step was re-applied to obtain a core feature set specific to the CWRU dataset, and the enhancement strengths based on confidence were recalibrated according to the signal distribution of the CWRU dataset.

The proposed model achieved an accuracy of 97.40%, a precision of 97.74%, a recall of 97.86%, an F1 score of 97.80%, and an AUC of 99.09%, with an average inference time of 0.0329 ms per sample. These results indicate that the proposed two-stage approach remains effective on an independent bearing dataset with multiple operating conditions.

A supplementary mixed SNR validation was further conducted to examine the performance of RF-EBM under noisy signal conditions. Noisy samples with different SNR levels were included in both the training and testing data. As shown in Table 9, RF-EBM maintained good performance under the noisy training and testing conditions considered in this experiment.

Based on the four operating conditions in the CWRU dataset, a further leave-one-operating-condition-out validation was conducted to examine the performance of the proposed approach under unseen speed and load conditions. In each round, all samples from one operating condition were held out as the test set, while samples from the other three operating conditions were used for training. As shown in Table 10, across the four tests with unseen operating conditions, the model achieved an average accuracy of 92.66%, an average F1 score of 93.47%, and an average AUC of 98.41%. These results suggest that the proposed approach maintains reasonable diagnostic performance under unseen speed and load conditions.

## 4. Conclusions

In conclusion, this study proposes a two-stage interpretable fault diagnosis approach. The proposed approach first uses EBM for feature selection to reduce feature redundancy and then introduces RF through residual learning to partly compensate for the prediction bias of EBM. EBM-based analysis and SHAP analysis are further used to interpret the diagnostic process from the primary model and the RF residual enhancer, respectively.

Experimental results on the SEU dataset show that the proposed EBM-based feature selection procedure enables RF-EBM to achieve a classification accuracy of 94.62% and an F1 score of 94.61%, improving traditional EBM by 2.13 and 2.16 percentage points, respectively. In the CWRU validation experiment, the proposed approach achieves an accuracy of 97.40% and an F1 score of 97.80%. The supplementary mixed SNR validation further shows that RF-EBM maintains good diagnostic performance under the tested noisy signal conditions. In the additional leave-one-operating-condition-out validation, the approach achieves an average accuracy of 92.66% and an average F1 score of 93.47% under unseen speed and load conditions. These results suggest that the proposed approach demonstrates good diagnostic performance and practical applicability under the tested laboratory benchmark conditions. Furthermore, the consistency between the EBM and SHAP feature importance rankings suggests that the selected core features can support a more reliable diagnostic interpretation. The shape function and SHAP analyses further illustrate how the core features influence the diagnostic results, improving the transparency of the decision-making process. Therefore, the proposed approach may provide a useful reference for interpretable bearing fault diagnosis by reducing feature redundancy, improving diagnostic performance, and supporting interpretable decision analysis.

The proposed method is primarily evaluated for bearing fault diagnosis under controlled operating scenarios with multiple relatively stable speed and load settings. The public laboratory benchmark datasets used in this study provide a basis for evaluating diagnostic performance and interpretability under such conditions. However, real industrial environments may involve stronger noise interference, load fluctuations, continuously varying speeds, and hardware deployment constraints. Therefore, future work will further validate the proposed method using real industrial data and investigate its robustness and deployment performance under more complex diagnostic conditions.

## Figures and Tables

**Table 1:** List of time-domain features.

Feature	Full Name	Physical Meaning
max	Maximum	Maximum signal value
min	Minimum	Minimum signal value
mean	Mean	Average signal level
peak	Peak-to-peak value	Difference between maximum and minimum values
arv	Average rectified value	Mean absolute amplitude
var	Variance	Dispersion of signal values
std	Standard deviation	Fluctuation of signal amplitude
kurtosis	Kurtosis	Impulsiveness of the vibration signal
skewness	Skewness	Asymmetry of amplitude distribution
rms	Root mean square	Energy level of the vibration signal
waveformf	Waveform factor	Ratio of RMS to average rectified value
peakf	Peak factor	Ratio of peak-to-peak value to RMS
impulsef	Impulse factor	Ratio of peak-to-peak value to average rectified value
clearancef	Clearance factor	Sensitivity to impact components

**Table 2:** List of frequency-domain features.

Feature	Full Name	Physical Meaning
fc	Frequency centroid	Weighted average frequency of the spectrum
msf	Mean square frequency	Average squared frequency of the spectrum
rmsf	Root mean square frequency	Square root of mean square frequency
vf	Frequency variance	Dispersion of frequency components
rvf	Root variance frequency	Spectral width of frequency components
ampmean	Amplitude mean	Mean spectral amplitude
ampstd	Amplitude standard deviation	Fluctuation of spectral amplitude
ampskewness	Amplitude skewness	Asymmetry of spectral amplitude distribution
ampkurtosis	Amplitude kurtosis	Peakedness of spectral amplitude distribution

**Table 3:** Bearing condition types in the SEU dataset.

Number	Type	Description
1	Ball	Crack occurs in the ball
2	Inner	Crack occurs in the inner ring
3	Outer	Crack occurs in the outer ring
4	Combination	Crack occurs in both inner and outer rings
5	Normal	No crack

**Table 4:** Main parameter settings of the EBM model.

Parameter	Value
Number of interactions	5
Maximum bins	64
Maximum rounds	150
Learning rate	0.03

**Table 5:** Random feature sets used for comparison with the core feature set.

Group	Feature 1	Feature 2	Feature 3	Feature 4	Feature 5
Random Group 1	ampstd	peak	max	kurtosis	rms
Random Group 2	rms	arv	peak	ampskewness	vf
Random Group 3	mean	rvf	clearancef	min	max
Random Group 4	mean	std	rms	rmsf	ampmean
Random Group 5	max	vf	std	ampkurtosis	ampstd

**Table 6:** Diagnostic performance comparison between random feature sets and the core feature set.

Feature Set	ACC (%)	F1 (%)
Random Group 1	90.84	90.76
Random Group 2	90.59	90.57
Random Group 3	88.31	88.31
Random Group 4	82.00	81.93
Random Group 5	88.07	88.67
**Ours**	**92.49**	**92.45**

**Table 7:** Diagnostic performance comparison of five models on the core feature set.

Model	Acc (%)	Prec (%)	Rec (%)	F1 (%)	AUC (%)	Infer_T (ms)
SVM	81.78	82.44	81.78	80.98	95.25	**0.0019**
KNN	93.53	93.53	93.53	93.52	98.70	0.0219
MLP	93.72	93.71	93.72	93.71	99.52	0.0737
XGBoost	94.57	94.58	94.57	94.56	**99.65**	0.0196
RF	**94.63**	**94.63**	**94.63**	**94.62**	99.62	0.0187

**Table 8:** Main parameter settings of the RF residual learner.

Parameter	Value
Number of decision trees	300
Splitting criterion	Gini index
Minimum samples for split	2
Minimum samples per leaf	1
Maximum features	Square root
Bootstrap sampling	Enabled
Random seed	42

**Table 9:** Diagnostic performance of RF-EBM under different SNR levels on the CWRU dataset.

SNR (dB)	Acc (%)	Prec (%)	Rec (%)	F1 (%)	AUC (%)	Infer_T (ms)
20	98.70	98.09	98.92	98.49	99.66	0.0655
15	98.65	98.37	98.85	98.60	99.63	0.0709
10	98.07	97.90	98.36	98.12	99.44	0.1533
5	97.55	97.15	98.00	97.56	99.22	0.0881
0	95.21	94.39	95.68	94.99	98.57	0.0852
−5	88.59	90.65	90.27	90.45	95.95	0.1323

**Table 10:** Diagnostic performance of RF-EBM under unseen speed and load conditions on the CWRU dataset.

Speed (rpm)	Load (HP)	Acc (%)	Prec (%)	Rec (%)	F1 (%)	AUC (%)	Infer_T (ms)
1730	3	91.25	93.29	91.65	91.80	97.39	0.0398
1750	2	98.50	98.68	98.88	98.77	99.66	0.0391
1772	1	92.00	93.87	92.51	93.09	97.95	0.0497
1797	0	88.88	92.67	89.44	90.21	98.62	0.0484

## Data Availability

Data and source code used in the paper can be accessed by contacting the authors.
